# Case Report: splenic abscess as a rare complication of perforated splenic flexure colorectal carcinoma: the role of imaging and multidisciplinary management

**DOI:** 10.3389/fonc.2026.1735936

**Published:** 2026-04-07

**Authors:** Qingfeng Chen, Xiaorui Liu, Aimin Zhang, Tao Zhang, Junjie Sun

**Affiliations:** 1 School of Clinical Medicine, Hebei University, Baoding, Hebei, China; 2 Department of Gastrointestinal Surgery, Affiliated Hospital of Hebei University, Baoding, Hebei, China

**Keywords:** colorectal carcinoma, colosplenic fistula, dual-pathway hypothesis, multidisciplinary team, splenic abscess

## Abstract

**Background:**

Splenic abscess is an exceedingly rare complication of colorectal carcinoma, particularly when associated with tumor perforation, colosplenic fistula, and multiorgan invasion.

**Case presentation:**

We present the case of a 71-year-old male patient with splenic flexure colorectal carcinoma (CRC) complicated by colosplenic fistula, splenic abscess, and invasion of the spleen, pancreas, and gastric wall, which were identified by contrast-enhanced computed tomography (CT). Following multidisciplinary team (MDT) consultation, an emergency laparotomy with multiorgan *en bloc* resection (left hemicolectomy, splenectomy, partial gastrectomy, distal pancreatectomy) achieved complete tumor removal.

**Conclusions:**

A novel “Dual-Pathway” Hypothesis is proposed to explain CRC-associated splenic abscess pathogenesis. A structured diagnostic and treatment workflow is presented to guide future management of such complex cases. This report highlights the critical roles of advanced imaging and MDT collaboration in optimizing outcomes for rare CRC complications.

## 1 Introduction

Colorectal cancer (CRC) is the third most common malignancy worldwide; however, splenic abscess remains an exceptionally rare and life-threatening complication, particularly when associated with tumor perforation and colosplenic fistula (1–3). The association between CRC and splenic abscess is underexplored. Existing literature comprises sporadic case reports, emphasizing mechanisms such as tumor infiltration into the splenic parenchyma or metastatic seeding via the portal circulation (4–6).

Diagnostic challenges persist due to nonspecific symptoms and imaging ambiguities. Contrast-enhanced CT is the gold standard for diagnosis, but distinguishing abscesses from necrotic tumors requires careful clinical correlation (6). Therapeutically, extensive disease often necessitates aggressive surgical intervention, guided by multidisciplinary team (MDT) expertise (7).

We report a rare case of splenic flexure CRC complicated by colosplenic fistula, splenic abscess, and multiorgan invasion. A proposed “Dual-Pathway” Hypothesis elucidates the pathogenesis and an MDT-guided workflow optimizes outcomes in these critical scenarios.

## 2 Case report

### 2.1 Clinical presentation

**Figure 1:**
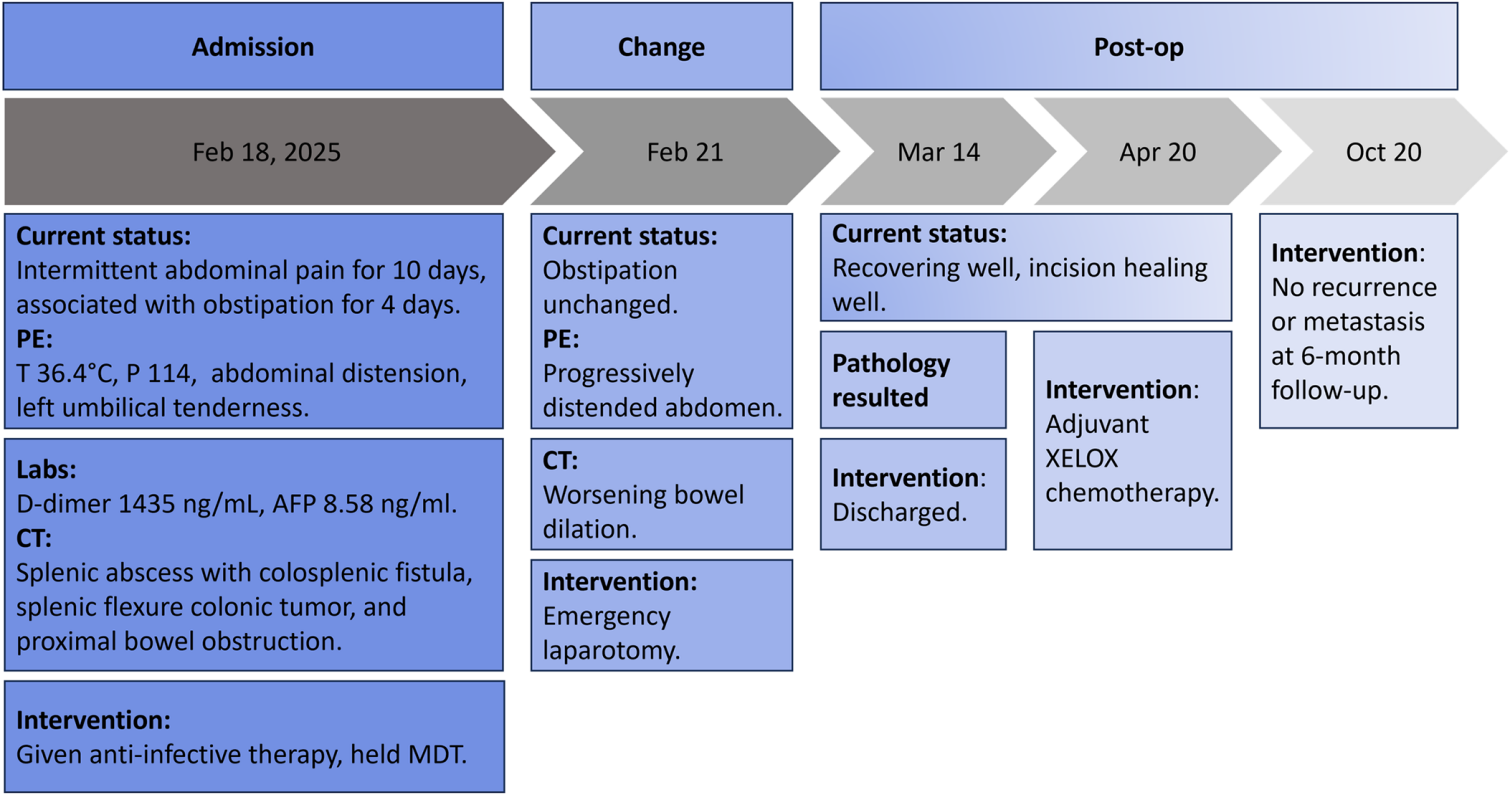
Timeline of clinical events and treatment from admission to six months post-surgery.

A 71-year-old male presented with 10 days of intermittent abdominal pain and distension, worsening over 4 days with cessation of flatus, defecation, nausea, and vomiting. He had no significant past surgical or family history, denied smoking or illicit drug use, and reported occasional alcohol consumption (3–4 drinks weekly). Physical examination revealed a temperature of 36.4°C, pulse of 114 beats/min, respiration of 20 breaths/min, and blood pressure of 133/89 mmHg. The abdomen was distended, soft, with tenderness in the left umbilical region but no rebound tenderness or palpable mass. A detailed clinical timeline is presented in **Figure 1**.

### 2.2 Laboratory and imaging findings

Laboratory tests revealed a normal white blood cell count (7.45 × 10^9^/L) but elevated D-dimer (1435.0 ng/mL). Tumor markers (CEA, CA19-9, CA72-4) were normal, with only a mildly elevated alpha-fetoprotein (AFP) of 8.58 ng/mL.

**Figure 2:**
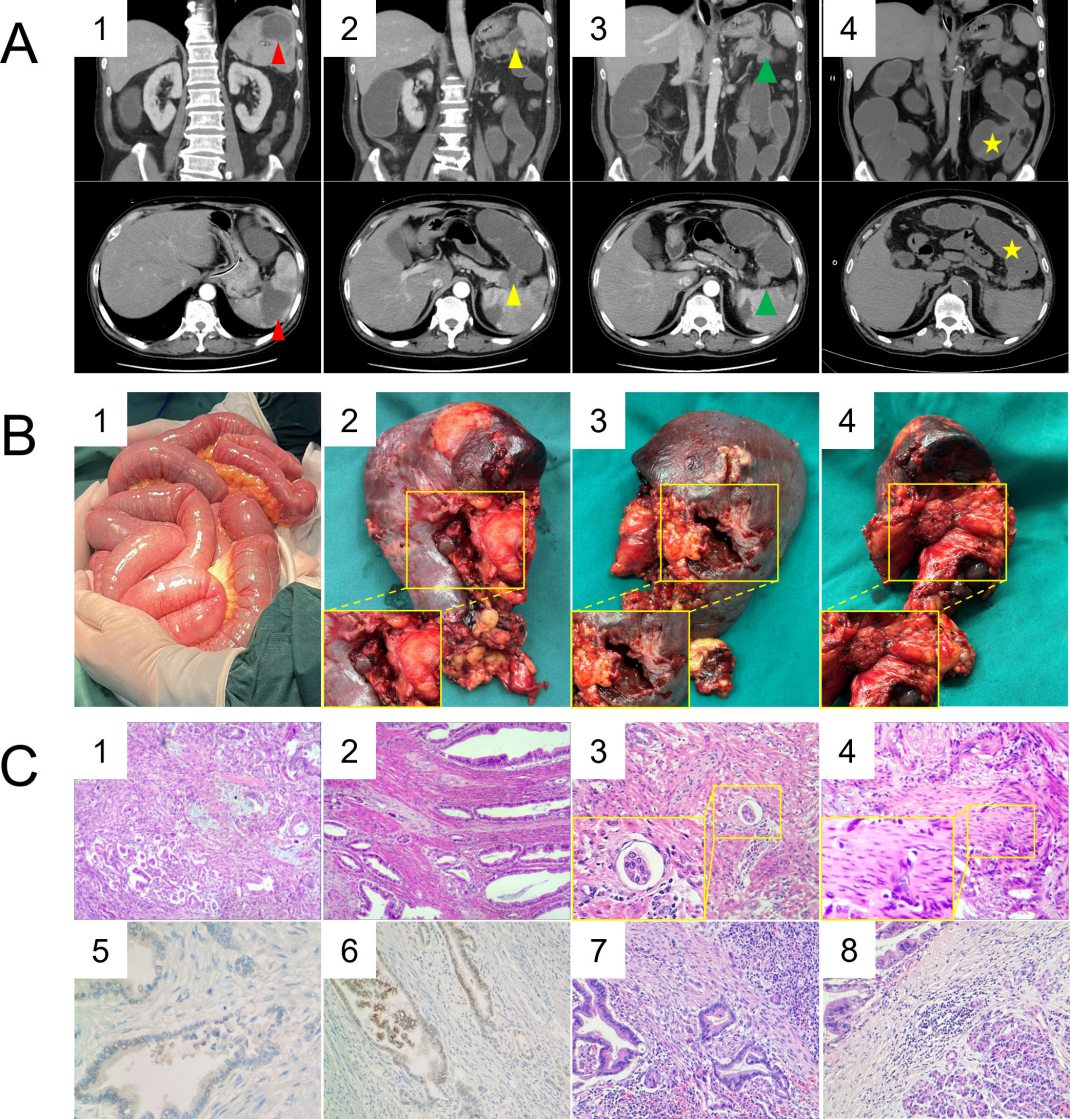
Radiologic-Pathologic Correlation. **(A)** CT images. **(A1)** Contrast-enhanced axial and coronal CT images (Feb 18, 2025) reveal multiple ill-defined, low-attenuation splenic lesions exhibiting peripheral rim enhancement (red triangle). **(A2)** Internal gas foci within the spleen track along the splenic hilum, demonstrating a suspected fistulous communication with the colonic splenic flexure (yellow triangle). **(A3)** The adjacent splenic flexure shows asymmetric mural thickening and heterogeneous enhancement with focal luminal narrowing (green triangle). **(A4)** Non-contrast CT (Feb 21, 2025) demonstrates progressive dilatation of the small bowel loops (yellow star). **(B)** Intraoperative findings. **(B1)** Significant small bowel dilation. (B2) A fistulous opening at the splenic hilum. (B3) A central splenic abscess cavity. (B4) Partial resection of pancreatic tail tissue. **(C)** Histopathological and immunohistochemical features. (C1) Moderately to poorly differentiated adenocarcinoma of the colon (H&E, 40×). (C2) Tumor infiltration extending through the full thickness of the colonic wall (transmural invasion) (H&E, 40×). (C3) Presence of intravascular tumor emboli (lymphovascular invasion) (H&E, 100×). (C4) Perineural invasion identified in the pericolic tissue (H&E, 100×). (C5) Immunohistochemical staining demonstrating partial nuclear positivity for CDX2 (IHC, 200×). (C6) Immunohistochemical staining showing positivity for CK20 (IHC, 200×). (C7) Metastatic adenocarcinoma in splenic tissue (H&E, 100×). (C8) Metastatic adenocarcinoma in pancreatic tail tissue (H&E,100×).

Contrast-enhanced abdominopelvic CT revealed a splenic abscess with colosplenic fistula (**Figures 2A1, A2**), a splenic flexure colonic tumor (**Figure 2A3**), and proximal bowel obstruction. A repeat non-contrast CT showed worsening bowel dilation (**Figure 2A4**).

### 2.3 MDT consultation and decision

Given the nonspecific clinical presentation and the presence of large bowel obstruction precluding endoscopic biopsy, preoperative diagnosis and treatment planning posed significant challenges. Thus, contrast-enhanced CT was critical for diagnosis. After integrated review of the clinical, laboratory, and imaging findings, the MDT including radiologists, oncologists, surgeons, and intensive care specialists, reached a consensus that there was a strong possibility of colonic tumor invading the spleen and pancreas, complicated by a splenic abscess. *En bloc* multiorgan resection was deemed the most appropriate therapeutic strategy to achieve both oncologic clearance and source control of the abscess. Due to the coexistence of a splenic flexure tumor and large bowel obstruction, percutaneous drainage was deemed insufficient as a definitive therapeutic strategy. It would fail to address the underlying perforation, potentially leading to persistent fecal contamination and tumor dissemination. As the malignant obstruction was refractory to conservative measures such as enema, and the patient showed no clinical improvement after three days of anti-infective therapy, emergency exploratory laparotomy was recommended.

### 2.4 Surgical intervention

Intraoperative findings revealed significant dilation of the small bowel (**Figure 2B1**), ascending colon, and transverse colon. A palpable mass, approximately 4×3×2 cm, was identified at the splenic flexure of the colon, which had perforated and was densely adhered to the spleen, forming a central splenic abscess cavity (**Figures 2B2, B3**) approximately 5 cm in diameter. The colonic mass was found to be intimately connected to the pancreatic tail and the spleen. Consequently, the patient underwent exploratory laparotomy combined with left hemicolectomy, splenectomy, distal pancreatectomy (**Figure 2B4**), and enterotomy for decompression. A sample of the splenic pus was collected and sent for bacterial culture. The surgery was completed successfully, and the patient was transferred to the recovery ward in stable condition.

### 2.5 Pathological outcomes

Postoperative pathology confirmed a moderately to poorly differentiated adenocarcinoma involving the left splenic flexure, infiltrating the full thickness of the colonic wall (**Figures 2C1, C2**). Histological features included focal lymphovascular invasion and perineural invasion (**Figures 2C3, C4**). Immunohistochemistry showed MLH1 (+), MSH2 (+), MSH6 (+), PMS2 (+), CDX2 (partial+) (**Figure 2C5**), CK20 (+) (**Figure 2C6**), CK7 (+), CA19-9 (+), CK19 (+), SATB-2 (–). The tumor directly invaded the pancreatic parenchyma and perisplenic adipose tissue (**Figures 2C7, C8**). The spleen demonstrated extensive necrosis with abscess formation, consistent with the preoperative diagnosis. All resection margins, including the proximal and distal colonic margins and the pancreatic resection margin, were free of tumor. Additionally, no metastasis was found in the omental tissue or pericolic lymph nodes (0/32). The final pathological stage was pT4bN0M0.

### 2.6 Patient perspective

From the patient’s perspective, the diagnostic process was marked by uncertainty and fear. Postoperatively, he expressed relief at the successful resection but also anxiety regarding recurrence and the need for adjuvant chemotherapy. This case shows that psychological support is essential in managing oncologic emergencies, alongside surgical intervention.

### 2.7 Postoperative course

The patient had an uneventful postoperative recovery and was discharged three weeks after surgery. Adjuvant XELOX chemotherapy began 1 month later, with no evidence of recurrence or metastasis at the 6-month follow-up.

## 3 Discussion

Splenic abscess secondary to CRC is exceptionally rare, with fewer than 20 cases documented (8). Prior reports illustrate varied clinical trajectories. Yelisetti et al. described tumor perforation with abscess but no fistula, managed by Hartmann’s procedure (9). Awotar et al. reported a colosplenic fistula treated by drainage alone without splenectomy; the patient subsequently developed metastases, underscoring the inadequacy of conservative management (8). Goldberg et al. documented a colosplenic fistula in suspected lymphoma, but the patient died without surgical intervention (10). Wadhwani and Diwakar reported a colocutaneous fistula managed with neoadjuvant chemotherapy and resection, yet the patient succumbed to postoperative infection (11). In contrast, the present case is the first to demonstrate the complete pathologic triad—colosplenic fistula, culture-positive splenic abscess, and multiorgan invasion—all confirmed histopathologically, with R0 resection achieved upfront and no recurrence at six months.

### 3.1 Pathogenesis of CRC-associated splenic abscess: the “dual-pathway” hypothesis

**Figure 3:**
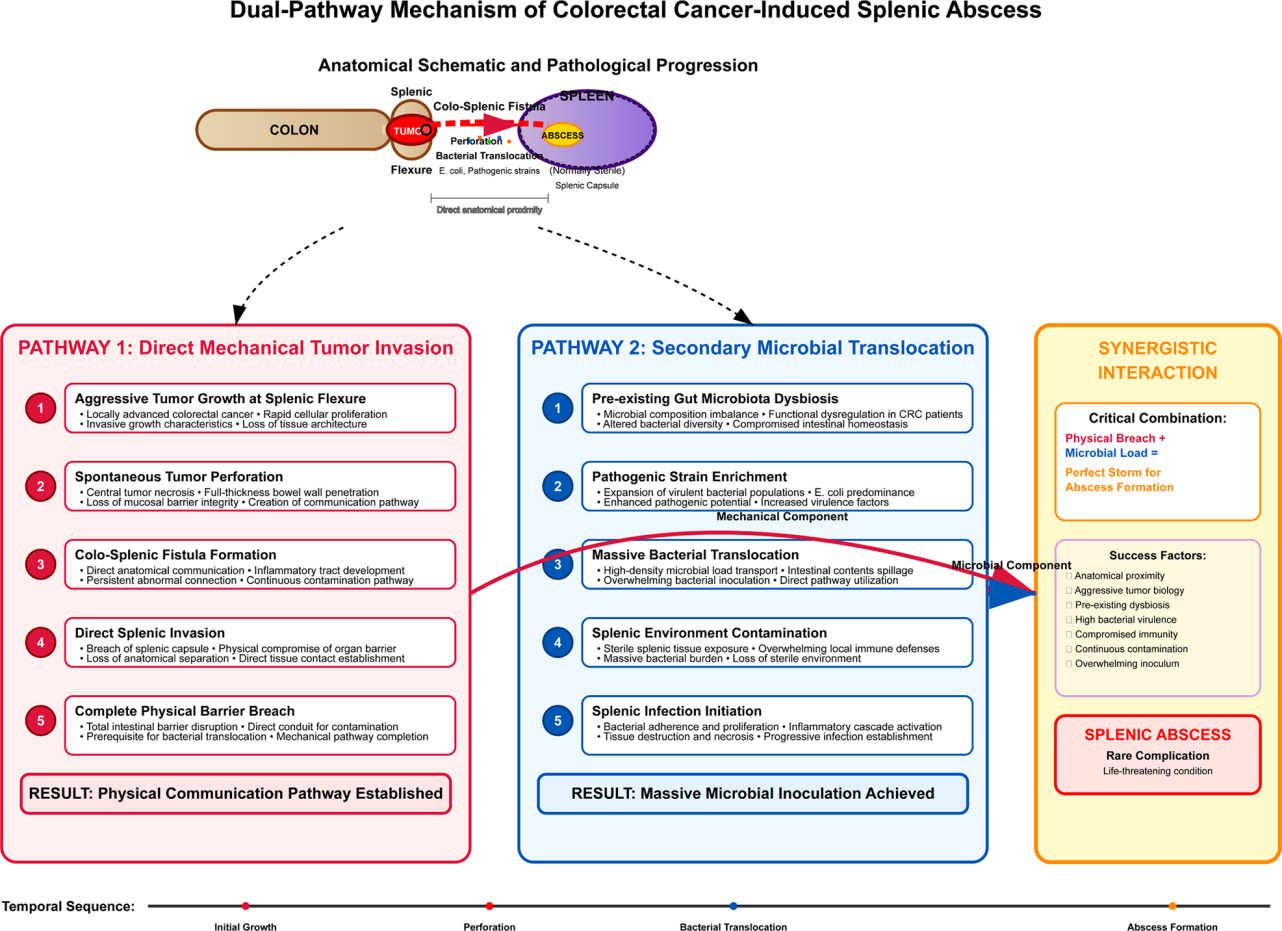
Schematic diagram illustrating the “Dual-Pathway” Hypothesis for CRC-associated splenic abscess.

While splenic abscesses in CRC patients have traditionally been attributed to either hematogenous seeding (via portal or systemic circulation) or direct contiguous invasion from the primary tumor, neither mechanism fully accounts for the constellation of findings in the present case. To elucidate the pathogenesis of such a rare complication, we propose a novel “Dual-Pathway” Hypothesis (**Figure 3**).

- Pathway 1: Mechanical Invasion. Aggressive tumor growth at the splenic flexure leads to perforation and the formation of a colosplenic fistula, breaching the intestinal barrier and allowing direct tumor infiltration into the spleen.
- Pathway 2: Microbial Translocation. Gut microbiota dysbiosis enables pathogenic bacteria (*e.g.*, *Escherichia coli*, as cultured in this case) to translocate via the fistula into the spleen, initiating abscess formation (10, 12–14).

This hypothesis integrates mechanical and microbiological mechanisms, supported by studies linking gut dysbiosis to CRC progression via toll-like receptor pathways and myeloid-derived suppressor cell recruitment (15–18). The novelty lies in the proposed synergistic coupling of two parallel pathways, rather than their isolated operation.

### 3.2 Synergistic role of contrast-enhanced CT and MDT in surgical decision-making

Contrast-enhanced CT is critical for diagnosis, providing the anatomical clarity essential for MDT-guided surgical planning (19–21). The MDT approach is pivotal (20, 22), facilitating: 1) Preoperative Planning: Radiologists provide anatomical insights for precise surgical strategies; 2) Intraoperative Decision-Making: Surgeons collaborate to achieve R0 resection via *en bloc* multiorgan resection and 3) Postoperative Management: Infectious disease specialists tailor antibiotics based on culture results. The MDT model ensured the scientific rigor and continuity of care and was central to optimizing the patient’s outcome.

### 3.3 Proposed workflow

**Figure 4:**
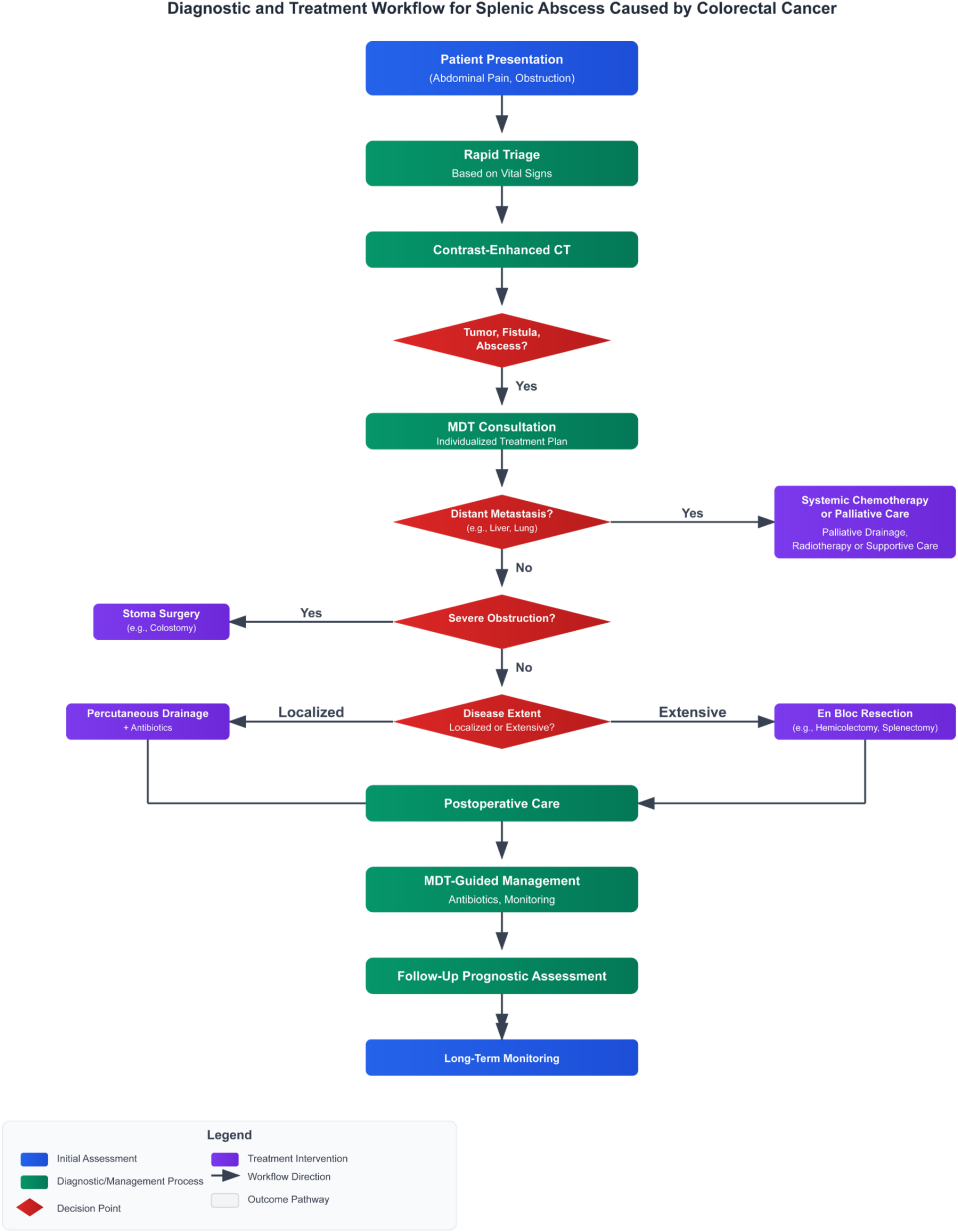
Flowchart of the proposed diagnostic and treatment workflow for CRC-associated splenic abscess.

Given the absence of standardized management guidelines, we propose a structured diagnostic and treatment workflow based on our experience (**Figure 4**). This workflow aims to provide decision support for future clinical practice, with its core principles including: Rapid Triage Based on Vital Signs, MDT-Led Individualized Plan Formulation, Long-Term Prognostic Assessment and Follow-up.

### 3.4 Limitations

As a single-case study, the generalizability of our findings is limited. Preoperative endoscopy was infeasible due to obstruction, potentially missing additional diagnostic insights. The resource-intensive MDT and surgical approach may not be feasible in low-resource settings (21), limiting the applicability of the proposed workflow. Long-term outcomes beyond 6 months are unknown, necessitating extended follow-up. Future multicenter studies are needed to validate the “Dual-Pathway” Hypothesis and workflow.

## 4 Conclusion

In summary, CRC complicated by splenic abscess is an extremely rare clinical entity, whose complex pathophysiological process can be reasonably explained by the “Dual-Pathway” Hypothesis. Contrast-enhanced CT is the cornerstone for accurate diagnosis and surgical planning. Given its high risk and complexity, curative surgical resection aimed at tumor eradication is the preferred treatment. Throughout the diagnostic and treatment process, a responsive and closely collaborative MDT is central to ensuring the best possible patient outcomes. The structured diagnostic and treatment workflow we propose can serve as a reference framework for managing similar challenging and critical cases in the future.

## Data Availability

The original contributions presented in the study are included in the article/**Supplementary Material**. Further inquiries can be directed to the corresponding authors.

## Glossary

CRC: colorectal carcinoma
MDT: Multidisciplinary Team
